# Group housing during adolescence has long-term effects on the adult stress response in female, but not male, zebra finches (*Taeniopygia guttata*)

**DOI:** 10.1016/j.ygcen.2017.07.008

**Published:** 2018-01-15

**Authors:** Michael G. Emmerson, Karen A. Spencer

**Affiliations:** University of St Andrews, School of Psychology & Neuroscience, St Mary’s Quad, South Street, St Andrews, Fife KY16 9JP, Scotland, United Kingdom

**Keywords:** Adolescence, Plasticity, Group size, Crowding, Social stress, Stress response

## Abstract

Adolescent social interactions can have long-term effects on physiological responses to stressors in later-life. A larger adolescent group size can result in higher stressor-induced secretion of glucocorticoids in adulthood. The effect may be due to a socially-mediated modulation of gonadal hormones, e.g. testosterone. However, group size (number of animals) has been conflated with social density (space per animal). Therefore it is hard to determine the mechanisms through which adolescent group size can affect the stress response. The current study aimed to tease apart the effects of group size and social density during adolescence on the physiological stress response and gonadal hormone levels in adulthood. Adolescent zebra finches were housed in groups varying in size (2 vs. 5 birds per cage) and density (0.03 m^3^ vs. 0.06 m^3^ per bird) during early adolescence (day 40–60). Density was only manipulated in birds raised in groups of five. Glucocorticoid concentration secreted in response to a standard capture and restraint stressor was quantified in adolescence (day 55 ± 1) and adulthood (day 100+). Basal gonadal hormone concentrations (male testosterone, female estradiol) were also quantified in adulthood. Female birds housed in larger groups, independent of social density, secreted a higher glucocorticoid concentration 45 min into restraint regardless of age, and had higher peak glucocorticoid concentration in adulthood. Adult gonadal hormone concentrations were not affected by group size or density. Our results suggest that group size, not density, is a social condition that influences the development of the endocrine response to stressors in female zebra finches, and that these effects persist into adulthood. The findings have clear relevance to the social housing conditions necessary for optimal welfare in captive animals, but also elucidate the role of social rearing conditions in the emergence of responses to stressors that may persist across the lifespan and affect fitness of animals in wild populations.

## Highlights

• Adolescent group size, not density, influences adult corticosterone secretion.
• A larger group size results in higher corticosterone secretion during restraint.
• The adolescent group size effects on corticosterone were only found in females.

## 1 Introduction

Stressors are unpredictable and uncontrollable stimuli that are perceived to be a (potential) threat (Koolhaas et al., 2011), such as being captured (Angelier et al., 2010, Romero and Reed, 2005) or separated from a social group (Hennessey, 1997, Remage-Healey et al., 2003). A well conserved characteristic of vertebrate species is an acute rise in glucocorticoid (GC) concentration in response to stressors, such as cortisol in primates and fish and corticosterone in birds and some rodents (Denver, 2009, Romero, 2004, Sapolsky et al., 2000). An acute rise in GCs is an adaptive strategy to cope with a short-term stressor as the hormones elicit risk-avoidant behaviors (Haller et al., 1998, Rodgers et al., 1999) that may promote successful avoidance of stressors (Ferrari et al., 2015a, Ferrari et al., 2015b). However, repeated or prolonged stress can cause chronic secretion of GCs leading to deleterious effects, such as suppression of reproduction, higher rates of programmed cell death, and immunosuppression (McEwen and Wingfield, 2003, McEwen, 1998, Toufexis et al., 2014). Chronically high GC concentrations may therefore impair fitness (Bonier et al., 2009, Breuner et al., 2008). For example, chronically high GC concentrations can result in birds engaging in higher rates of nest abandonment (Love et al., 2004, Ouyang et al., 2012) and raising fewer offspring (Thierry et al., 2013). Chronic secretion of stress hormones also contributes ‘wear and tear’ to systems affected by GCs, e.g. cardiovascular system (McEwen, 1998, McEwen and Wingfield, 2003), and can induce oxidative stress (Constantini et al., 2011) that may in turn lower survival prospects through higher disease susceptibility, as documented in rats (*Rattus norvegicus*: Cavigelli and McClintock, 2003, Cavigelli et al., 2009). The acute physiological stress response therefore has clear consequences for fitness and animal welfare, emphasising the need to further determine the factors that cause variation in GC secretion.

The social interactions an animal engages in during development can have immediate and sustained effects on GC secretion (Lukkes et al., 2009, Sachser et al., 2013, Weintraub et al., 2010). Adolescence is the developmental stage spanning from puberty to sexual maturity (Brown and Spencer, 2013, Sisk and Foster, 2004, Spear, 2000) and is a crucial developmental stage for social interactions as adolescents begin to interact more with age-similar conspecifics and integrate into larger social groups (Gunnar and Hostinar, 2015, Nelson et al., 2005, Spear, 2000). Animals living in larger groups engage in more social interactions with one another compared to animals living in smaller groups (Van Loo et al., 2001, Judge and de Waal, 1993), so variation in group size can be used to investigate how the quantity of social interactions can modulate GC secretion. For example, adolescent male guinea pigs (*Cavia porcellus*) housed in larger groups have a lower GC secretion in response to single housing in an unfamiliar environment compared to conspecifics reared in smaller groups (Sachser et al., 1993, Sachser et al., 2013). However, group size has also been shown to have no short-term effect on GC secretion in mice (*Mus musculus*: Ago et al., 2014, Laviola et al., 2002). Also in mice, adolescent group size has no long-term effects on basal GC concentration (Ortiz et al., 1985). However, mice raised in larger, vs. smaller, groups in adolescence had a higher GC concentration in response to a loud noise when single housed in adulthood (Ortiz et al., 1985). Higher GC levels in response to a stressor when alone may reflect greater perception of threat when separated from conspecifics (Ortiz et al., 1985). Perhaps a higher number of adolescent social interactions results in adult animals that are more reliant on conspecifics for coping with stressors, with higher GC levels in those raised in larger groups possibly resulting in behaviors that re-establish social contact with a group for protection (Hawkley et al., 2012). However, this hypothesis requires testing.

Basal concentrations of gonadal hormones rise as adolescence progresses, resulting in a higher basal concentration of testosterone in males and estradiol in females (Delemarre-van-de-Waal, 2002, Sisk and Foster, 2004). Testosterone can inhibit, whilst estradiol can stimulate, GC secretion (McCormick and Mathews, 2007, Green and McCormick, 2016); an effect that emerges in adolescence, at least for testosterone in males (Foilb et al., 2011, Gomez et al., 2004, Hennessey et al., 2002, Romeo et al., 2004). Social interactions stimulate testosterone secretion (Eisenegger et al., 2011, Lürzel et al., 2011), with adolescent male guinea pigs housed in large groups having higher basal testosterone concentration than conspecifics raised in smaller groups (Sachser and Prӧve, 1988, Lürzel et al., 2010). Animals may engage in more social interactions in larger groups, resulting in a testosterone-mediated inhibition of GC secretion (Hennessey et al., 2009, Sachser et al., 2013). However, adolescent group size has no effect on basal testosterone concentration in adolescent mice (Laviola et al., 2002, Smith et al., 2004) or adult mice (Nicholson et al., 2009, Ortiz et al., 1984, Smith et al., 2004), with further studies necessary to determine if the effects of adolescent group size on testosterone are specific to guinea pigs. How female acute stress responses are regulated by gonadal hormones, such as estradiol, across animals raised under different adolescent housing conditions remains to be investigated. Nonapeptides also regulate social interactions (Goodson and Thompson, 2010) and the acute stress response (Lightman, 2008), potentially acting as an alternative mechanism in the social regulation of GC secretion. Vasopressin (in mammals) and vasotocin (in birds) stimulate GC secretion during a stress response (Aguilera and Rabadan-Diehl, 2000, Scott and Dinan, 1998), while oxytocin (in mammals) and potentially mesotocin (in birds) inhibit GC secretion (Goodson et al., 2015, Neumann, 2008). The effect of adolescent social experiences on nonapeptide concentrations remains to be determined.

A higher number of social interactions during adolescence, facilitated by housing in a larger group, is known to elevate testosterone in adolescence. This might in turn lower GC secretion in response to stressors during adolescence. However, a higher number of social interactions in adolescence may also result in animals developing into adults that are more reliant on conspecifics for coping with stressors. This might in turn heighten stressor-induced GC secretion in adulthood when separated from conspecifics. However, most studies on which these inferences are based have achieved a larger group size by housing more animals per cage (e.g. Sachser et al., 1993, Ortiz et al., 1985) thereby conflating group size and social density effects. Social density may facilitate social interactions beyond that observed in those living in larger groups due to a restriction in available space per animal (e.g. van Loo et al., 2001). In addition, the effects of adolescent group size and social density have largely been investigated in males (e.g. Ago et al., 2014, Sachser et al., 1993), and whether sex differences occur has not been adequately tested. Male and female mice have been shown to have similar basal GC concentrations regardless of social density (Laviola et al., 2002) and, like adult males, higher social density in adult females results in higher basal GC concentration in mice (e.g. Ishida et al., 2003) and chickens (*Gallus gallus domesticus*: Craig and Swanson, 1994, Kang et al., 2016). Variation in group size and/or density has similar effects on GC concentrations in males and females, so sex differences in response to variation in adolescent group size and/or social density would not be expected, but still require direct investigation.

The current study aimed to investigate the short- and long-term effects of adolescent group size and density on the acute stress response. Zebra finches (*Taeniopygia guttata*), a passerine bird that flocks in groups that vary widely in number and density of birds (Griffith and Buchanan, 2010, Zann, 1996), were used. Zebra finches undergo adolescence from postnatal days 30 to 100, with sexual maturation occurring at a similar age in both sexes (Zann, 1996). During early adolescence (around day 50), the birds begin to spend more time interacting with unfamiliar conspecifics instead of parents (Adkins-Regan and Leung, 2006, Zann, 1996). Early adolescence may therefore be a time when the birds are more influenced by age-similar conspecifics than at other times in adolescence. Previous work has also shown that zebra finches are more influenced by variation in GC exposure during early adolescence (days 40–60) compared to late adolescence (days 65–85), further indicating early adolescence as an age to investigate social contexts that may cause variation in GC concentrations (Emmerson and Spencer, 2017). Housing zebra finches in groups of two or six birds during adolescence has long-term effects on social behavior (Ruploh et al., 2014), but endocrine effects of such housing variations have not yet been explored. Birds in the current study were housed in groups that varied in number (2 vs. 5 birds per cage) and density (0.03 m^3^ vs. 0.06 m^3^ per bird) during days 40–60. Corticosterone (CORT), the GC that is secreted in response to a stressor in zebra finches, was quantified in response to a standard capture and restraint stressor in adolescence (day 54–56) and adulthood (day 100+). Basal concentration of gonadal hormones (male testosterone, female estradiol) were also quantified in adulthood. Birds raised in larger (vs. smaller) and denser (vs. less dense) conditions were predicted to have lower CORT concentration in response to capture and restraint in adolescence, but higher CORT concentration in response to capture and restraint in adulthood (based on Ortiz et al., 1985, Sachser et al., 1993). Basal gonadal hormone concentrations were predicted to be no different between adolescent housing conditions (based on, for example, Nicholson et al., 2009). No differences between males and females in GC concentrations were expected to occur (based on Laviola et al., 2002).

## 2 Materials and methods

### 2.1 Ethical statement

All ethical guidelines and requirements, as set out in the Principles of Laboratory Animal Care (NIH, Publication No. 85–23, revised 1985) and the UK Home Office Animals (Scientific Procedures) Act 1986, were adhered to under project licence 70/8159 and personal licences IDFA58352, IEBE43CFF, and 60/13261.

### 2.2 Establishing the experimental population

The zebra finches used in this study (n = 76) were the offspring from 24 breeding pairs selected from an in-house breeding stock. To select which breeding birds would be housed together, the adult birds were housed in one of two mixed-sex colony cages (100 × 50 × 50 cm, length × height × depth; n = 12 male, 12 female per colony cage) that contained four nest boxes. Over a ten day period, hay nesting material was provided daily and observations were made in order to quantify courtship and copulatory behavior (e.g. following, directed song, mounting, sharing a nest box). Birds that were observed engaging in any of these behaviors on two separate days were removed from the breeding colony cages and housed together in individual breeding cages (60 × 50 × 50 cm, length × height × depth; MB 3612 Metal Double Breeding Cage, R.J. Leigh Ltd., UK) with access to a cardboard nest box (14 × 11 × 11 cm, height × length × depth). The breeding pairs had ad libitum access to one seed hopper (Food for Finches, Johnson & Jeff, UK), one water hopper, one water bath, and one grit tray at all times. Spinach leaves were provided once per week. Individual breeding cages contained two 50 cm perches and the cage floor was covered with wood pellets (Stovies Wood Pellets, Arbuthnott Wood Pellets Ltd, UK). All birds (breeding pairs and offspring) were housed in a single colony room throughout the experiment with lights on 07:00–19:00, temperature 20–24 °C, and relative humidity 50–60%.

Each breeding pair was provided with new nesting material (hay and jute, Liverine Pet and Animal Health Care Ltd., UK) once per day until the birds had laid a clutch. Fresh egg food (approx. 1.5 g of CéDé Premium Egg Food, Belgium) was also provided to the pairs once per day until the chicks reached nutritional independence (35 days old). Eggs were removed from the nest on the day they were laid, and then replaced with a fake egg (Staedtler Fimo Soft Oven Hardened Modelling Clay (white), UK). Once a female had stopped laying (no new eggs on two consecutive days) the fake eggs were removed and the real eggs were replaced. Egg removal and replacement was carried out in order to synchronise hatching and control for any hatch order effects (Mainwaring and Hartley, 2013). Each clutch was candled on incubation day 7 in order to determine fertility of the eggs. Any clutches that were infertile had eggs removed in order to allow relaying. On the first day of hatching, brood sizes were standardised to control for variation in pre-adolescent group size and social density. As a brood of four chicks was the modal brood size in the current experiment (20/24 nests), broods were standardised to four chicks. Nests with more than four chicks (2/24) were reduced to four by placing excess chicks in donor nests that were not used, whilst nests with fewer than four chicks (2/24) were not used. On postnatal day 10, chicks were provided with a permanent ID (one uniquely numbered orange leg ring and one coloured leg ring: pink, yellow, light blue, or white). Parents were removed from each breeding cage at day 35.

### 2.3 Experimental design

**Fig. 1:**
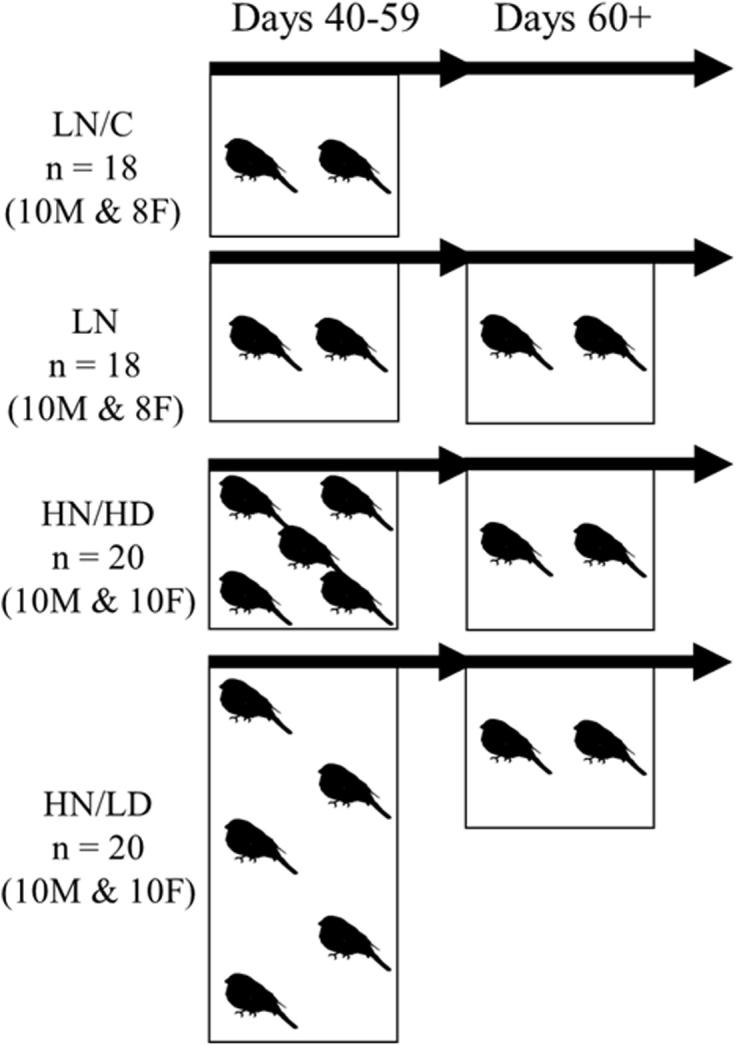
Summary diagram of housing conditions that birds in the current study underwent during early adolescence. During days 40–59 low number (LN), low number/control (LN/C), and high number/high density (HN/HD) birds were housed in cages measuring 60 × 50 × 50 cm (length × height × depth) whereas high number/low density (HN/LD) birds were housed in larger cages measuring 120 × 50 × 50 cm (length × height × depth). On day 60 LN/C birds were re-housed with a familiar cage mate, whereas LN, HN/LD, and HN/HD birds were pair housed with an unfamiliar conspecific from the same group. Birds were kept in same-sex groups throughout the experiment.

Between postnatal days 40 and 59, the birds were continuously housed in same-sex and age-similar (+/− 1 day) housing conditions that varied in group size and social density (Fig. 1). Housing conditions were termed low number (LN), low number/control (LN/C), high number/low density (HN/LD), and high number/high density (HN/HD). On day 60, variation in group size and density were ended by re-housing all birds in same-sex age-similar (+/− 1 day) pairs in cages measuring 0.6 × 0.5 × 0.5 m (length × height × depth). LN, HN/LD, and HN/HD birds were re-housed with an unfamiliar bird from a different replicate of the same housing condition. LN/C birds were captured and then re-housed with their familiar cage mate. Adolescent social novelty can lower GC secretion in adult rats (Caruso et al., 2014), so LN/C birds can be compared to LN, HN/LD, and HN/HD birds to investigate any potential effects of social novelty on adult endocrine measures.

To investigate social density, birds in HN/LD and HN/HD conditions were housed five birds per cage (n = 20 per condition, 10 male and 10 female, split into four replicates of five birds). HN/LD replicates were housed in cages measuring 1.2 × 0.5 × 0.5 m (length × height × depth; 0.06 m^3^ per bird), whilst HN/HD replicates were housed in cages measuring 0.6 × 0.5 × 0.5 m (length × height × depth; 0.03 m^3^ per bird). To investigate group size, birds housed in groups of five (HN/LD & HN/HD) could be compared to birds housed in groups of two (LN & LN/C; n = 18 per condition, 10 male and 8 female, split into 9 replicates of two birds) in cages measuring 0.6 × 0.5 × 0.5 m (length × height × depth). LN and LN/C replicates each had access to one seed hopper, one water hopper, one water bath, and one grit tray. Birds in HN/LD and HN/HD replicates had access to two of each hopper, two water baths, and two grit trays in an attempt to standardise resources across group sizes. Birds in LN, LN/C, and HN/HD conditions had access to two 50cm perches, but birds in the HN/LD condition had access to four 50cm perches so they could occupy more space.

### 2.4 Hormone sampling

The concentration of CORT in response to a standard capture/restraint stressor (Wingfield and Romero, 2001) was quantified in adolescence (postnatal day 54–56) and adulthood (postnatal day 147–177). At each age, three samples were collected in order to quantify the basal CORT concentration and two stressor-induced CORT concentrations. Birds were captured from their home cages and taken to a separate room for blood sampling. All blood samples were taken by pricking the brachial vein with a 27-gauge needle tip. Blood was then collected in heparinised capillary tubes before being transferred to an Eppendorf on wet ice. To ensure an accurate basal CORT concentration the first blood sample (approx. 40 µl) was collected within three minutes of entering the holding room (Romero, 2004). After the first sample was taken the birds were restrained in black cloth bags, with a second blood sample (approx. 30 µl) taken fifteen minutes after entering the holding room and a third blood sample (approx. 30 µl) taken forty-five minutes after entering the holding room. After the final blood sample was taken all birds were returned to their home cages with their familiar cage mates. All birds from a home cage were captured and sampled at the same time to control for any effect of cage disturbance. Blood samples were centrifuged at 3500*g* for ten minutes to separate the plasma from the red blood cells, after which the plasma was extracted and stored at -20 °C. Later in adulthood (day 166–196), a further blood sample (approx. 40 µl) was taken from each bird in order to determine the basal concentration of testosterone in males and estradiol in females. This blood sampling protocol was identical to that for collecting a basal sample of CORT, but with all samples collected within four minutes of entering the holding room to ensure that a basal testosterone sample was collected (Wingfield and Wada, 1989). Plasma was extracted and stored identically to that described for the CORT samples.

### 2.5 Hormone assays

#### 2.5.1 Corticosterone

The CORT concentration in samples of plasma (10–30 µl) from all birds were quantified via radioimmunoassay (Spencer et al., 2009). The samples were first extracted with 1 ml diethyl ether after being spiked with 25 µl of [1,2,6,7-3H]-CORT label (Perkin Elmer Inc., UK). The extracted samples were evaporated at 42 °C and reconstituted in 300 µl of assay buffer (0.01 M PBS, pH 7.4, 0.25% BSA). Extraction efficiency was quantified from a 50 µl aliquot taken from each of the reconstituted samples and ranged between 71.24 and 100%. CORT concentration was determined in 100 µl aliquots of the extracted samples. The assays were performed using anti-CORT antiserum (Esoterix Endocrinology, USA, B3-163; 1:15,000 dilution in assay buffer) and [1,2,6,7-3H]-CORT label (Perkin Elmer, UK). Bound and free portions were separated using 500 µl of a dextran coated charcoal suspension (0.25% dextran, 0.5% charcoal). A total of six assays were performed. All samples from a single individual were run in duplicate in the same assay, with the different adolescent housing conditions and sexes distributed across the assays. Each assay included a ten point standard curve ranging from 0.04 to 20 ng/ml. Plasma samples of known concentrations (5, 10, and 20 ng/ml) were included in duplicate in each assay to determine intra- and inter-assay co-efficient of variation. Intra-assay coefficients of variation (%) were 10.51, 9.74, 12.86, 10.12, 11.37, and 13.15. Inter-assay coefficient of variation (%) was 12.70. 50% binding (ng/ml) values were 0.79, 0.74, 0.80, 0.76, 0.92, and 0.81. Detection limit was 0.04 ng/ml.

#### 2.5.2 Testosterone

The testosterone concentration in 20–30 µl samples of plasma from male birds was determined using radioimmunoassay. The testosterone assay was performed identically to that described for CORT (see Section 2.5.1 Corticosterone), but with the use of anti-testosterone antiserum (MP Biomedicals, LLC., USA, 07–189016) and [1,2,6,7-3H]-testosterone label (Perkin Elmer, UK). All samples were run in duplicate in a single assay. Extraction efficiency ranged between 75 and 100%, intra-assay co-efficient of variation was 5.34%, 50% binding was 0.39 ng/ml, and the detection limit was 0.04 ng/ml.

#### 2.5.3 Estradiol

The estradiol concentration was quantified using an enzyme immunoassay kit and following the manufactures guidelines (Cayman Chemical Company, Estradiol EIA Kit, Ann Arbor, Michigan, USA). The kit has previously been used to quantify estradiol in zebra finches (Remage-Healey et al., 2008, Remage-Healey et al., 2012). The samples of plasma (10–30 µl) were diluted in assay buffer to a final volume of 105 µl, with 50 µl aliquots run in duplicate on a single plate. The plate was read on a Biochrom Anthos 2010 Microplate Reader, ADAP 2.0 (Biochrom Ltd., UK) at a wavelength of 405 nm. Intra-assay coefficient of variation was 9.74% and the detection limit was 6.6 pg/ml.

### 2.6 Data analysis

All statistical analyses were conducted using SPSS v.22. The residuals from each model were checked for normality, with any variables that were not normally distributed (Shapiro-Wilk, p < 0.05) transformed to reach normality. Individual ID and nest ID were entered as random factors in any mixed models to control for inter-individual differences and pre-adolescent experiences, respectively. Log10 CORT concentration (ng/ml) was entered as a dependent variable in a linear mixed model with housing condition, sampling time, age, and sex entered as fixed factors (main effects and interactions). Sampling time and age were also entered as repeated measures. Step-wise deletion of non-significant terms was used to simplify the model investigating CORT concentration over sampling times. The following four interactions were removed from the full model: housing condition × age, housing condition × age × sex, housing condition × age × sampling time, and housing condition × age × sampling time × sex. To further analyse the response to capture/restraint, an analysis was conducted on peak CORT concentration (i.e. the largest CORT value for each individual at either 15 or 45 min sampling times). Log10 peak CORT concentration (ng/ml) was entered as a dependent variable in a linear mixed model with housing condition, age, and sex entered as fixed factors (main effects and interactions). Age was entered as a repeated measure. Basal CORT concentration was entered as a co-variate in the peak CORT model to control for differences in initial CORT concentration. For gonadal hormones, square root estradiol concentration (pg/ml) and log10 testosterone concentration were entered as dependent variables in separate linear mixed models. Adolescent housing condition was entered as a fixed factor in both gonadal hormone models. Significant effects were investigated with Sidak and Bonferroni post hoc tests for independent and repeated measures, respectively. To further assess the link between CORT and gonadal hormones Spearman’s rank correlations were conducted. Separate models were conducted for each sex and age group combination with basal, 15 min, 45 min, and peak CORT values for adolescent and adult samples entered as dependent variables in each model alongside either testosterone (male models) or estradiol (female models). An alpha value of 0.05 was used as the threshold for statistical significance in LMMs, but a Bonferroni corrected alpha value of 0.0016 was applied for correlational analyses to correct for multiple (n = 32) comparisons. Cohen’s d was calculated as a measure of effect size between significant post hoc comparisons. All data presented are means +/− standard error of the mean.

## 3 Results

Only the most complex significant interactions are presented below in order to provide a clearer report of the data. A full output from each model can be found in the Supplementary data.

### 3.1 CORT response to capture and restraint over time

**Fig. 2:**
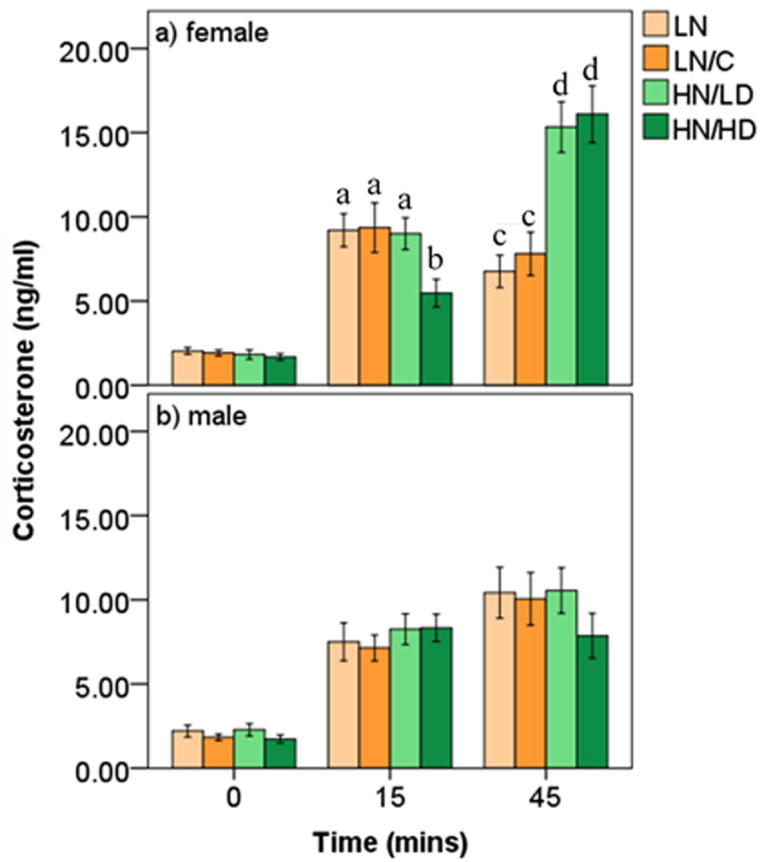
Corticosterone concentration (ng/ml) secreted by zebra finches in response to a capture and restraint stressor averaged across sampling ages (adolescence and adulthood) and split by sampling time (0, 15, 45 mins), adolescent housing condition (LN, LN/C, HN/LD, HN/HD), and sex (female, male) with a) showing female values and b) showing male values. Significant differences between housing conditions within a single sex and a single time sampling time are denoted by a vs. b, and c vs. d (p < 0.05).

In the time-response CORT concentration model an interaction between housing condition, sampling time, and sex was significant (F_6,68.001_ = 7.835, p < 0.001; Fig. 2a/b). The interaction was present regardless of age (F_6,68_ = 1.569, p = 0.170). 15 min into restraint, CORT concentrations of HN/HD females were significantly lower than those of female birds from all other conditions (LN vs. HN/HD, p = 0.027, d = 0.81; LN/C vs. HN/HD, p = 0.012, d = 0.99; HN/LD vs. HN/HD, p = 0.020, d = 0.89). 45 min into restraint, female birds that were housed in larger groups (regardless of density) exhibited higher CORT levels than those housed in smaller groups (LN vs. HN/LD, p = 0.014, d = 1.24; LN vs. HN/HD, p = 0.012, d = 1.27; LN/C vs. HN/LD, p = 0.007, d = 1.52; LN/C vs. HN/HD, p = 0.006, d 1.53). All comparisons between male conditions were not significant at 15 min (p’s > 0.534; Supplementary Table S1) and 45 min (p’s > 0.346; Supplementary Table S1).

### 3.2 Peak CORT response to capture and restraint

**Fig. 3:**
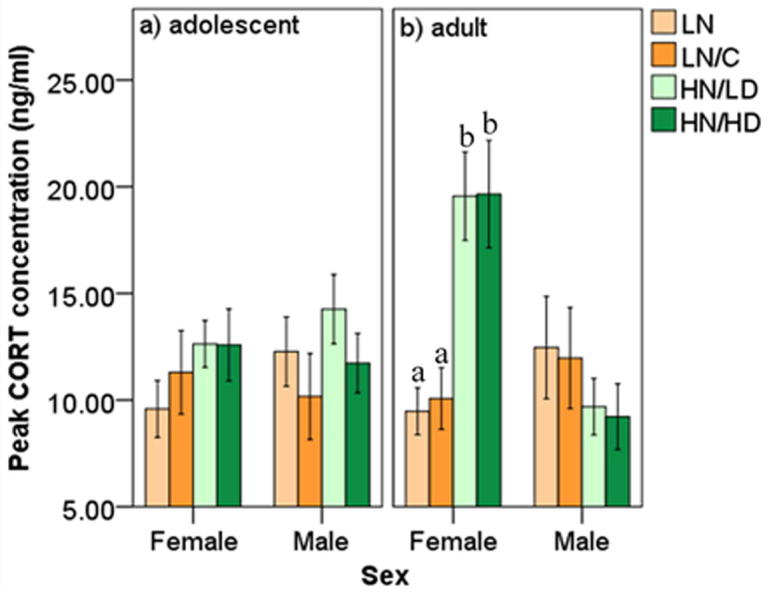
Peak concentration (ng/ml) of corticosterone (CORT) secreted by zebra finches in response to a capture and restraint stressor split by sampling age (adolescent and adulthood), sex (female and male), and adolescent housing condition (LN, LN/C, HN/LD, HN/HD). Any significant differences between housing conditions within a single sex and a single age are denoted by a vs. b (p < 0.05).

In the peak CORT concentration model an interaction between housing condition, sex, and age was significant (F_3,68.152_ = 5.386, p = 0.002; Fig. 3a/b). In adolescent females, peak CORT concentrations were no different across housing conditions (LN v. LN/C, p = 0.892; LN vs. HN/LD, p = 0.734; LN vs. HN/HD, p = 0.792; LN/C v. HN/LD, p = 0.994; LN/C vs. HN/HD, p = 0.831; HN/LD vs. HN/HD, p = 0.884). In adult females, birds that were housed in large groups in early adolescence had a peak CORT concentration that was higher than that of birds housed in small groups (LN vs. HN/LD, p = 0.022, d = 1.70; LN vs. HN/HD, p = 0.028, d = 1.46; LN/C vs. HN/LD, p = 0.013, d = 1.90; LN/C vs. HN/HD, p = 0.018, d = 1.61). Within female comparisons across ages revealed that peak CORT concentration of birds housed in the larger groups was higher in adulthood compared to adolescence (HN/LD, p = 0.009,d = 1.33; HN/HD, p = 0.003, d = 1.04), but no age differences were found in female birds housed in smaller groups (LN, p = 0.691; LN/C, p = 0.952). In males, peak CORT concentrations were no different across housing conditions in either adolescence (p’s > 0.308; Supplementary Table S2) or adulthood (p’s > 0.672; Supplementary Table S2). Age comparisons within each male housing condition revealed that peak CORT concentration was higher in adolescent compared to adult HN/LD birds (p = 0.015, d = 1.03), but all other age comparisons were not significant (LN/C, p = 0.281; LN, p = 0.702; HN/HD, p = 0.281). All birds in each housing condition had a similar peak CORT concentration in adolescence (LN/C, p = 0.547; LN, p = 0.366; HN/LD, p = 0.637; HN/HD, p = 0.883). In adulthood, female birds that were housed in larger groups in early adolescence had a higher peak CORT concentration than that of adult male birds housed in the same way (HN/LD, p = 0.001, d = 1.80; HN/HD, p < 0.001, d = 1.58). No sex difference was found between adult birds housed in the smaller groups (LN, p = 0.652; LN/C, p = 0.558).

### 3.3 Adult basal concentration of gonadal hormones

The basal testosterone concentration in adult males was not affected by adolescent housing condition (F_3,36_ = 0.181, p = 0.908; Table 1) and the basal estradiol concentration in adult females was not affected by adolescent housing condition (F_3,32_ = 0.187, p = 0.905; Table 1).

**Table 1:** Estradiol concentration (pg/ml) of adult female zebra finches and testosterone concentration (ng/ml) of adult male zebra finches split by adolescent housing condition (LN/F, LN/U, HN/LD, HN/HD). No significant differences were found between housing conditions within a single sex (p > 0.05).

Gonadal hormone	Adolescent housing condition			
	LN	LN/C	HN/LD	HN/HD
Estradiol (pg/ml)	51.38 (11.08)	50.13 (12.68)	39.02 (6.00)	48.00 (12.13)
Testosterone (ng/ml)	1.36 (0.43)	1.58 (0.39)	1.42 (0.45)	1.15 (0.26)

### 3.4 Correlations between CORT and gonadal hormones

Adult basal testosterone concentration was not correlated with any CORT concentration within adolescent males (p’s > 0.022) or adult males (p’s > 0.117). Adult basal estradiol concentration was similarly not correlated with any CORT concentration in adolescent females (p’s > 0.233) or adult females (p’s > 0.047). A full report of all correlations can be found in the Supplementary data (male correlations, Table S3; female correlations, Table S4).

## 4 Discussion

The findings from the current study clearly show that the size of the social group an animal belongs to during adolescence can have persistent effects on the physiological stress response, but in a sex-dependent manner. Female birds housed in larger groups in early adolescence secreted a higher concentration of GCs in response to an acute stressor; an effect that emerges in adolescence and persists into adulthood. The higher GC response in larger groups during adolescence was unexpected, as prior studies find no effect of adolescent group size on adolescent GC concentrations (e.g. Laviola et al., 2002). A higher GC response in adults from larger adolescent groups was in line with previous research (e.g. Ortiz et al., 1985). However, group size and density were conflated in previous work that has varied group size by housing more animals per cage without changing cage size. The current study attempted to separate the effect of group size from social density, and suggests that only early adolescent group size affects CORT secretion as the social density variation between large group reared birds had little effect on CORT secretion. Early adolescent social network size may therefore be a variable used by female zebra finches to modulate acute physiological responses to stressors.

### 4.1 Adolescent group size effects on stressor-induced CORT secretion over time

Female birds that were housed in groups of five during early adolescence had a higher CORT concentration 45 min into restraint compared to female birds housed in groups of two during early adolescence. The adrenal glands may therefore continue to secrete CORT into the general circulation for a longer duration of time in birds raised in the larger groups compared to smaller groups. However, as the sampling times used in the current study did not capture all aspects of a stress response (i.e. no post-peak CORT concentration) it cannot be ruled out that the females raised in larger groups had a delayed or slower rise in CORT over time compared to females raised in smaller groups. The cause of the effects of adolescent group size on female birds’ CORT response is yet to be elucidated. However, variation in central and/or peripheral regulation of the stress response (i.e. the hypothalamic-pituitary-adrenal axis) may be responsible. Excitation of the paraventricular nucleus of the hypothalamus (PVN) stimulates a stress response (Herman et al., 2003), with higher PVN activity associated with higher GC secretion (e.g. Romeo et al., 2006). Adrenocorticotropic hormone (ACTH) binds to MC2 receptors in the adrenal gland to trigger the secretion of GCs (Herman et al., 2003), with higher adrenal MC2 receptor expression associated with higher stressor-induced GC concentration (e.g. Romeo et al., 2014). Higher PVN activity and/or higher adrenal expression of ACTH receptors could cause higher GC secretion. However, a lower adrenal MC2 receptor expression could lower sensitivity to ACTH resulting in delayed CORT secretion. Further work is necessary to test the hypotheses that either central and/or peripheral changes in stress regulation occur in response to adolescent group size, and by doing so may reveal whether adolescent group size effects the quantity or duration of stressor-induced CORT secretion in female zebra finches.

Adolescent group size has no effect on GC secretion in response to social separation alone (Lürzel et al., 2010), but a larger adolescent group size does heighten GC secretion in response to a loud noise when socially separated (Ortiz et al., 1985) and when restrained whilst socially separated as shown in the current study. GC hormones elicit risk-avoidant behavior (Haller et al., 1998, Rodgers et al., 1999), which in affiliative species can include locating conspecifics for group protection (Hawkley et al., 2012). Consequently, birds raised in larger groups may have a higher stress response when alone as part of a more risk-avoidant phenotype, with the birds attempting to seek out social support when individually exposed to a stressor. The stress response (physiological and behavioral) of animals raised in different adolescent group sizes would need to be quantified in both the presence and absence of conspecifics to test this hypothesis. Adolescent group size could improve fitness in wild populations of social animals due to more risk-avoidance (e.g. Ferrari et al., 2015a). However, welfare could be impaired by larger adolescent group size in low stress environments (e.g. lab housing) as the advantages of higher GC secretion (e.g. risk-avoidance: Haller et al., 1998) are not pertinent whilst animals still accumulate the costs of higher GC secretion (e.g. oxidative damage, Constantini et al., 2011; shorter lifespan, Cavigelli et al., 2009). Further work is therefore necessary to determine the role of adolescent group size both for individual fitness in wild populations and in the determination of the conditions for optimal lab animal welfare. For example, female zebra finches housed in larger groups during early adolescence may be expected to have a greater incidence of disease and shorter longevity (i.e. poorer welfare) compared to birds raised in smaller groups when the birds are raised under captive conditions, but the hypothesis requires testing.

### 4.2 Age-specific effects of adolescent group size on CORT secretion

Peak CORT concentration in response to capture and restraint revealed that the effects of adolescent group size were dependent on age. In adulthood, female birds that were housed in larger groups during early adolescence had a higher peak CORT concentration than other birds. However, group size during adolescence had no immediate effect on peak CORT. The discrepancy of age-related effects on measures of CORT between the time response and peak CORT models may reflect that different aspects of the stress response were analysed. However, the time response model was more complex than that the peak CORT model. A simpler peak CORT model may therefore have more statistical power and better detect age-related effects, thereby providing a more precise representation of the effects of adolescent group size. Adolescent social interactions therefore have long-term effects on the physiological response to stressors in adulthood as has previously been reported (Lukkes et al., 2009, Sachser et al., 2013, Weintraub et al., 2010). The age-specific effect indicates that PVN activity and/or adrenal gland sensitivity do not simply reflect the adolescent social context. Instead, the adolescent social context may influence the ontogeny of PVN and/or adrenal responses to stressors that do not fully emerge until adulthood. Investigating these neuroendocrine responses to stressors (PVN activity, adrenal sensitivity, & GC secretion) across development in animals reared in different social conditions (like group sizes) is necessary to determine the precise age at which the higher stress response emergences and why.

Finding that adolescent group size has no immediate effect on GC secretion has corroboration from previous work in mice and guinea pigs that also show that adolescent group size has no immediate effect on basal GC concentration (Ago et al., 2014, Laviola et al., 2002, Ortiz et al., 1985, Sachser et al., 1993; but not Lürzel et al., 2010). Our work expands on this prior research to suggest that the absence of effect is not due to a limited scope of measurement (i.e. basal concentration only). Instead, adolescent group size appears to have no short-term effect on the acute stress response. However, studies quantifying the short-term effects of group size on GC concentration have sampled animals between 9 and 29 days into the variation in social housing. Consequently, no study has quantified GCs immediately or shortly after being housed in larger or denser groups. Animals may initially respond to higher social density by engaging in more antagonistic behavior, but over time antagonistic behavior is supplanted by more affiliative and submissive behavior (Judge and de Waal, 1993, Judge and de Waal, 1997) as they learn to cope with living in larger or denser groups (de Waal, 1989). The lack of group size and social density effects on peak CORT (and basal CORT in previous work) may therefore reflect that animals have had sufficient time to cope with living in a larger or denser social group. It would therefore be advantageous to quantify GC concentrations at different time points after housing adolescent animals in larger and/or denser social groups to account for any time-dependent effects.

### 4.3 Why did adolescent group size have female-specific effects on CORT secretion?

The effects of adolescent group size were limited to female birds, with male birds being unaffected by either adolescent group size or social density. Why only female birds are affected is not clear, but the sex-dependent effects could be due to age-related changes in the response to stressors and/or social context. Adolescent female zebra finches (day 60) have a higher basal and peak CORT concentration in response to capture and restraint compared to age-similar male conspecifics (Crino et al., 2014), but this effect may be limited to birds from smaller broods (Spencer et al., 2009). The standardised brood size in the current study rules out any effect of brood size, but the reported effects emphasise that the social context during development can influence sex differences in CORT secretion. Future work may aim to investigate the social housing context by quantifying social behavior during the adolescent manipulation (days 40–60) to see how these behaviors relate to the acute stress response. The mechanism by which female, but not male, birds use group size to modulate stress physiology remains elusive in part because most research investigating adolescent group size effects on GC secretion have only used males (e.g. Ago et al., 2014, Lürzel et al., 2010, Lürzel et al., 2011, Ortiz et al., 1985, Sachser et al., 1993). The single study that has included both males and females found no sex-dependent short-term effects of group size on CORT secretion in adolescent mice and did not look for long-term effects (Laviola et al., 2002). The findings we present in the current study are therefore the first to report the long-term effect of variation in adolescent group size and density on the GC response of adult females. These sex-specific effects emphasise the importance for more research to include both male and female animals to further understand how the social environment during development can have sex-specific effects on life history strategies and optimal animal welfare.

Adolescent male and female zebra finches undergo adolescence during a similar age range (postnatal days 30 and 100) and have similar rates of pubertal development for gonadal maturation and the emergence of adult-typical beak colour and plumage (Perfito, 2010, Zann, 1996). The sex-specific effects of early adolescent group size can therefore not be attributed to sex differences in pubertal maturation, for example with male and female birds in different stages of development during days 40–60. However, sex differences are present for some aspects of pubertal maturation in late adolescence. For example, from day 75 onwards male zebra finches have a higher basal testosterone concentration than female zebra finches (Zann, 1996). In adulthood, higher basal testosterone concentration results in more antagonistic interactions between male zebra finches (Ardia et al., 2010). Male zebra finches may therefore engage in more antagonistic interactions than females during late adolescence. A larger and/or denser group can result in more antagonistic interactions compared to smaller and/or less dense groups in males of some rodent species (e.g. Van Loo et al., 2001) that can result in a higher CORT concentration (Creel et al., 2013). A larger and/or denser late adolescent group housing may therefore result in a greater secretion of stressor-induced CORT in male zebra finches compared to males raised in smaller and/or less dense groups in late adolescence. The current study therefore needs to be replicated, but during a late adolescent period (e.g. days 65–85: Emmerson and Spencer, 2017) to test this hypothesis.

### 4.4 Why did adolescent social density have little effect on CORT secretion?

Our novel approach to investigating the effects of group size and density revealed that adolescent social density had little effect on the acute stress response. Females raised in large groups at high density had lower CORT concentration at 15 min into restraint compared to all other housing conditions, indicating that social density may have some impact on CORT secretion. However, the high density effect may be an anomaly due to conflating CORT concentrations across ages when reporting the sex by sampling time interaction in the model used in the current study; the effect did not appear separately at any one age. Animals typically engage in more antagonistic interactions at higher social densities (e.g. van Loo et al., 2001) that would be expected to result in higher CORT concentration compared to animals raised at lower densities (Creel et al., 2013). The social density used in the current study may not have affected antagonistic behavior during adolescence, with no effects therefore found on CORT concentration. Alternatively, higher social density may result in animals engaging in more interactions of a type that may not influence the ontogeny of GC responses (e.g. submissive behavior: Judge and de Waal, 1997). Further research is necessary to determine if the variation in social density used in the current study affected social interactions during early adolescence to link the different housing conditions to effects on CORT. The design used in the current study was advantageous by including LN/C birds to investigate the effects of adolescent social novelty; a variable that has been shown to lower GC secretion in adult rats (Caruso et al., 2014). However, the design resulted in the effects of density not being fully explore as social density was only varied in large group reared birds. The current study therefore needs to be replicated using a 2 × 2 design that crosses group size with density to explore the effect of social density in low number groups before any firm conclusions can be made as to whether early adolescent social density truly has little effect on CORT secretion.

### 4.5 Adolescent group size and density effects on basal gonadal hormone concentrations

Basal concentrations of gonadal hormones in adulthood were not affected by group size and/or social density in early adolescence. In addition, the testosterone and estradiol concentrations did not consistently correlate with any measure of CORT. The absence of an effect of adolescent group size on adult male basal testosterone concentration is corroborated by prior research (Nicholson et al., 2009, Ortiz et al., 1984, Sachser et al., 1993, Smith et al., 2004), but the current study is the first to also show that basal estradiol concentration in adult females is similarly unaffected by early adolescent group size and/or social density. Concentrations of gonadal hormones can rise in response to stressor exposure (Wingfield and Wada, 1989, Yilmaz, 2003); a response that emerges in adolescence, at least for testosterone in males (Foilb et al., 2011, Romeo et al., 2004). The current study focused only on basal gonadal hormone concentration, but adolescent group size and/or social density could have influenced the dynamic response of gonadal hormones to stressors (Sachser et al., 1993). The current study could therefore be replicated with the intent to determine the effects of adolescent group size and/or social density on the secretion of gonadal hormones in response to stressors. Alternatively, group size may modulate GC secretion through changes in nonapeptide concentration. A higher CORT concentration secreted in response to restraint in adult animals that were housed in larger groups during adolescence may be due to a lower concentration of nonapeptides that inhibit GC secretion (e.g. oxytocin, mesotocin: Neumann, 2008, Goodson et al., 2015) and/or a higher concentration of nonapeptides that further stimulate GC secretion during a stress response (e.g. vasopressin, vasotocin: Aguilera and Rabadan-Diehl, 2000, Cornett et al., 2013). These hormones were not quantified in the current study, however. Further work is necessary to determine the effects of adolescent social experiences on adult measures of nonapeptide functioning to elucidate the potential role for these hormones in regulation of the adult acute stress response.

### 4.6 Conclusion

In the present study, we have clearly shown that female zebra finches housed in larger groups during early adolescence secrete a higher concentration of the stress hormone CORT when subjected to capture and restraint in adulthood. Due to our novel design, our findings are the first to specify that group size, not social density, is a factor resulting in later-life changes in CORT secretion. The findings suggest that social interaction quantity or social network size in adolescence act as cues to induce long-term effects on adult GC secretion, but the effects were only present in females. The long-term, and potentially life-long, alterations in the acute stress response have clear importance for determining the developmental conditions necessary for optimal welfare in laboratory animals and fitness of animals living in wild populations.
