# Supplementary material for: Group housing during adolescence has long-term effects on the adult stress response in female, but not male, zebra finches (Taeniopygia guttata)
Source: Gen Comp Endocrinol. 2018 Jan 15;256:71–9. doi: 10.1016/j.ygcen.2017.07.008 (PMC5771470; doi:10.1016/j.ygcen.2017.07.008)
Supplement: Supplementary data 1 [file mmc1.docx]

**Supplementary Data**

1. *CORT response to capture and restraint over time*

Stressor-induced CORT concentrations changed across the sampling times (F_2,67.897_ = 302.684, p < 0.001). Basal CORT concentration was lower than both the later time points (15 min: p < 0.001, d = 1.907; 45 min: p < 0.001, d = 1.77) and the CORT concentration at 15 mins was higher than at 45 mins (p = 0.008, d = 0.48). Housing condition had no effect on CORT concentration by itself (F_3,68.001_ = 1.707, p = 0.174), but housing condition did affect CORT concentration at specific sampling times (F_6,68.001_ = 2.724, p = 0.020). Basal concentrations of CORT in all groups were lower than at 15 mins (p’s < 0.001, d’s > 1.79) and 45 mins (p’s < 0.001, d’s > 1.55). In addition, birds housed in larger groups had lower CORT concentrations at 45 min than at 15 mins (HN/LD, p = 0.007, d = 0.80; HN/HD, p = 0.001, d = 0.76), whereas the concentrations of CORT at 15 and 45 mins were not significantly different between birds housed in smaller groups (LN, p = 0.832; LN/C, p = 0.764).

CORT concentration secreted in response to capture/restraint did not differ according to a bird’s age (F_1,74_ = 3.210 p = 0.077), but age effects did emerge at different sampling times (F_2,74_ = 24.289, p < 0.001). No age difference in basal CORT concentration was found (p = 0.644), but adolescents had higher CORT concentration than adults at 15 mins (p < 0.001, d = 0.83), and adults had higher CORT concentration than adolescents at 45 mins (p = 0.002, d = 0.42). Basal CORT concentration was lower than at the later time points in both adolescence (15 min: p < 0.001, d = 2.42; 45 min: p < 0.001, d = 1.85) and adulthood (15 min: p < 0.001, d = 1.62; 45 min: p < 0.001, d = 1.81). In adolescence CORT concentration was no different between 15 and 45 mins (p = 0.335), whereas in adults CORT concentration at 15 mins was higher than at 45 mins (p < 0.001, d = 0.95). An interaction between age and housing condition was not significant (F_3,71_ = 2.316, p = 0.083), and neither was an interaction between age, housing condition, and sampling time (F_6,71_ = 0.580, p = 0.745).

CORT concentration secreted in response to capture and restraint did not differ according to a bird’s sex (F_1,68.307_ = 0.585, p = 0.447). The interaction between sex and housing condition was also not significant (F_3,68.001_ = 0.070, p = 0.976), and neither were the interactions between sex and sampling time (F_2,67.897_ = 1.295, p = 0.281) and sex and age (F_1,74_ = 0.945, p = 0.334). However, sex-dependent age differences in CORT concentration did emerge at specific sampling times (F_2,74_ = 7.378, p = 0.001). No age differences were found in basal CORT concentration in both females (p = 0.168) and males (p = 0.429), adolescents had higher CORT concentration than adults at 15 mins in both females (p < 0.001, d = 0.64) and males (p < 0.001, d = 1.04), and at 45 mins the adult female birds had higher CORT concentration than adolescent females (p < 0.001, d = 0.88) but no age difference was found in males (p = 0.510). Sex-dependent effects on CORT concentration were not affected by housing condition and age (F_3,68_ = 1.778, p = 0.160).

1. *Peak CORT response to capture and restraint*

Peak CORT concentration in response to capture and restraint did not differ according to the birds’ adolescent housing condition (F_3,68.219_ = 2.229, p = 0.093), age (F_1,67.564_ = 0.306, p = 0.582) or an age by housing condition interaction (F_3,67.590_ = 0.224, p = 0.879). In addition, peak CORT concentration did not differ according to a bird’s sex (F_1,68.074_ = 2.350, p = 0.130) or an interaction between sex and housing condition (F_3,67.983_ = 2.068, p = 0.113). However, a significant interaction between a bird’s age and sex was found for peak CORT concentration (F_1,67.605_ = 10.013, p = 0.002). During adolescence there was no sex difference in peak CORT concentration (p = 0.747), but in adulthood female birds had higher peak CORT concentration than adult males (p = 0.005, d = 0.64). Adult female peak CORT concentration was also higher than the adolescent female peak CORT concentration (p = 0.013, d = 0.58), but there was no difference in peak CORT concentration between adolescent and adult males (p = 0.061).

1. *Tables for non-significant post hoc tests*

Table S1. Post hoc comparisons between male housing conditions for CORT concentration at 15 and 45 min into restraint. All data presented are p values from Bonferroni post hoc tests.

| Comparison | Time | |
| --- | --- | --- |
|  | 15 min | 45 min |
| LN vs. LN/C | 0.541 | 0.822 |
| LN vs. HN/LD | 0.781 | 0.621 |
| LN vs. HN/HD | 0.707 | 0.707 |
| LN/C vs. HN/LD | 0.656 | 0.783 |
| LN/C vs. HN/HD | 0.534 | 0.534 |
| HN/LD vs. HN/HD | 0.692 | 0.346 |

Table S2. Post hoc comparisons between male housing conditions for peak CORT concentration split by age. All data presented are p values from Bonferroni post hoc tests.

| Comparison | Age | |
| --- | --- | --- |
|  | Adolescent | Adult |
| LN vs. LN/C | 0.472 | 0.672 |
| LN vs. HN/LD | 0.541 | 0.829 |
| LN vs. HN/HD | 0.322 | 0.932 |
| LN/C vs. HN/LD | 0.308 | 0.749 |
| LN/C vs. HN/HD | 0.568 | 0.811 |
| HN/LD vs. HN/HD | 0.409 | 0.684 |

1. *Correlations between CORT and gonadal hormones*

Table S3. Correlations between adult basal testosterone and CORT concentrations in response to capture and restraint in adolescent and adult males. P values for all correlations were greater than the Bonferroni corrected α value of 0.0016.

| Age | CORT Variable | Testosterone | | | | | | | |
| --- | --- | --- | --- | --- | --- | --- | --- | --- | --- |
|  |  | LN | | LN/C | | HN/LD | | HN/HD | |
|  |  | r | p | r | p | r | p | r | p |
| Adolescent | 0 min | -0.006 | 0.987 | -0.709 | 0.022 | -0.067 | 0.865 | 0.260 | 0.441 |
|  | 15 min | 0.236 | 0.511 | 0.127 | 0.726 | -0.433 | 0.244 | -0.409 | 0.212 |
|  | 45 min | 0.285 | 0.425 | -0.588 | 0.074 | 0.450 | 0.224 | -0.164 | 0.631 |
|  | Peak | 0.383 | 0.275 | -0.079 | 0.829 | 0.100 | 0.798 | -0.355 | 0.285 |
| Adult | 0 min | 0.042 | 0.907 | -0.527 | 0.117 | 0.159 | 0.683 | 0.383 | 0.245 |
|  | 15 min | 0.115 | 0.751 | 0.394 | 0.260 | -0.417 | 0.265 | -0.182 | 0.593 |
|  | 45 min | 0.370 | 0.293 | -0.006 | 0.987 | 0.067 | 0.865 | -0.036 | 0.915 |
|  | Peak | 0.309 | 0.385 | 0.055 | 0.881 | 0.167 | 0.668 | -0.227 | 0.502 |

Table S4. Correlations between adult basal estradiol and CORT concentration in response to capture and restraint in adolescent and adult females. P values for all correlations were greater than the Bonferroni corrected α value of 0.0016.

| Age | CORT Variable | Estradiol | | | | | | | |
| --- | --- | --- | --- | --- | --- | --- | --- | --- | --- |
|  |  | LN | | LN/C | | HN/LD | | HN/HD | |
|  |  | r | p | r | p | r | p | r | p |
| Adolescent | 0 min | -0.167 | 0.693 | -0.311 | 0.453 | -0.103 | 0.776 | 0.333 | 0.347 |
|  | 15 min | -0.190 | 0.651 | -0.143 | 0.736 | 0.274 | 0.444 | -0.103 | 0.777 |
|  | 45 min | -0.452 | 0.260 | -0.476 | 0.233 | 0.018 | 0.960 | 0.212 | 0.556 |
|  | Peak | -0.190 | 0.651 | -0.143 | 0.736 | 0.158 | 0.663 | 0.212 | 0.556 |
| Adult | 0 min | -0.119 | 0.779 | -0.072 | 0.866 | -0.097 | 0.789 | 0.139 | 0.701 |
|  | 15 min | 0.714 | 0.047 | 0.071 | 0.867 | -0.055 | 0.881 | 0.055 | 0.881 |
|  | 45 min | -0.429 | 0.289 | -0.286 | 0.493 | -0.085 | 0.824 | 0.224 | 0.533 |
|  | Peak | -0.214 | 0.610 | -0.048 | 0.911 | -0.055 | 0.881 | 0.224 | 0.533 |
